# Should we measure clonal circulating plasma cells in light chain amyloidosis?

**DOI:** 10.18632/oncotarget.26289

**Published:** 2018-11-02

**Authors:** Surbhi Sidana, Shaji K. Kumar, Wilson I. Gonsalves

**Affiliations:** Division of Hematology, Department of Internal Medicine, Mayo Clinic, Rochester, MN, USA

**Keywords:** light chain, amyloidosis, circulating plasma cells, clonal plasma cells, myeloma

Light chain (AL) amyloidosis is typically characterized by low levels of clonal bone marrow plasma cells (BMPCs). However, the deposition of misfolded light chains produced by these cells leads to organ dysfunction with significant morbidity and mortality. It is well established that high levels of involved serum free immunoglobulin light chains and high BMPC burden (>10%) are poor prognostic markers in AL amyloidosis, along with advanced cardiac involvement [1, 2]. However, the variability in prognosis persists despite the currently used risk stratification systems. Hence, there is a need to study additional prognostic markers.

Clonal circulating plasma cells (cPCs) are an important biomarker in plasma cell disorders and their presence has been shown to have an adverse prognosis in monoclonal gammopathy of undetermined significance (MGUS) [3], as well as multiple myeloma (MM) [4–6]. Previously, cPCs were evaluated by plasma cell labeling index (PCLI), a slide-based technique. Evaluation of cPCs is now done by multi-parametric flow cytometry (MFC). In AL amyloidosis, cPCs have been demonstrated to have an adverse prognosis using slide based approaches, but this methodology is cumbersome and not widely adopted [7]. On the other hand, flow cytometry is widely available and easier to perform. We recently reported a retrospective study evaluating the prognostic significance of cPCs at the time of diagnosis using six color MFC (with antibodies to CD19, CD45, CD38, CD138, kappa and lambda) in 154 patients with AL amyloidosis (including those with overlapping symptomatic myeloma) from 2008-15 [8].

Among the study population, 42% of patients were found to have cPCs at diagnosis. The median number of CPCs was 81 per 150,000 events. Some patients can have an overlap of AL amyloidosis and myeloma with CRAB features (hypercalcemia, renal failure, anemia and bone disease). After excluding patients with CRAB features secondary to MM, 36% of patients were noted to have cPCs, with median value of 62 per 150,000 events.

Disease characteristics which may predict for the presence of cPCs were also evaluated. The percentage of BMPCs correlated with presence of cPCs, as did the presence of CRAB features and high levels of involved serum free immunogoublin light chains at diagnosis. However, only BMPCs were found to be an independent predictor of cPCs in multivariable analysis. Patients who had cPCs at diagnosis had inferior survival outcomes, both for overall survival (OS) and progression free survival (PFS). Estimated OS at 1, 2, and 5 years in patients with cPCs was: 74, 64, and 57% and in those without cPCs was 89, 87, and 80%, respectively (*p* = 0.003). cPCs were observed to be prognostic for OS in the sub-group of patients excluding CRAB features as well. Importantly, presence of cPCs remained an independent prognostic factor for OS (hazard ratio 2.1, 95% CI: 1.1-4.01, *p* = 0.03) after adjusting for other known prognostic factors (age, Mayo 2012 stage, 10% or more BMPCs). However, it is important to note that for patients achieving a very good partial response or better with therapy, the presence of cPCs at diagnosis was not prognostic, highlighting that achieving a deep response may overcome this adverse prognostic feature.

Thus, presence of cPCs can provide valuable prognostic information in AL amyloidosis at the time of diagnosis. It is therefore reasonable to evaluate for cPCs in patients with this disorder for risk stratification. If patients who have cPCs at diagnosis do not achieve a deep hematologic response, more frequent monitoring should be considered. Whether increasing cPCs levels have a proportional effect remains to be studied. Other areas of future investigation include evaluating the impact of early clearance/persistence of cPCs with treatment and whether the levels of cPCs impact outcomes in relapsed disease as well.
